# p53 stabilisation potentiates [^177^Lu]Lu-DOTATATE treatment in neuroblastoma xenografts

**DOI:** 10.1007/s00259-023-06462-3

**Published:** 2023-10-12

**Authors:** Hanna Berglund, Sara Lundsten Salomonsson, Tabassom Mohajershojai, Fernando Jose Ferrer Gago, David P. Lane, Marika Nestor

**Affiliations:** 1 Department of Immunology, Genetics and Pathology, Rudbeck Laboratory, Uppsala University, SE-751 85 Uppsala, Sweden; 2 Ridgeview Instruments AB, SE-752 38 Uppsala, Sweden; 3 p53Lab, Agency for Science Technology and Research (A*STAR), Singapore, 138648 Singapore; 4 Department of Microbiology, Tumour and Cell Biology, Karolinska Institute, SE-171 65 Solna, Sweden

**Keywords:** Neuroblastoma, Molecular radiotherapy, p53, [^177^Lu]Lu-DOTATATE, Radiosensitisation

## Abstract

**Purpose:**

Molecular radiotherapy is a treatment modality that is highly suitable for targeting micrometastases and [^177^Lu]Lu-DOTATATE is currently being explored as a potential novel treatment option for high-risk neuroblastoma. p53 is a key player in the proapoptotic signalling in response to radiation-induced DNA damage and is therefore a potential target for radiosensitisation.

**Methods:**

This study investigated the use of the p53 stabilising peptide VIP116 and [^177^Lu]Lu-DOTATATE, either alone or in combination, for treatment of neuroblastoma tumour xenografts in mice. Initially, the uptake of [^177^Lu]Lu-DOTATATE in the tumours was confirmed, and the efficacy of VIP116 as a monotherapy was evaluated. Subsequently, mice with neuroblastoma tumour xenografts were treated with placebo, VIP116, [^177^Lu]Lu-DOTATATE or a combination of both agents.

**Results:**

The results demonstrated that monotherapy with either VIP116 or [^177^Lu]Lu-DOTATATE significantly prolonged median survival compared to the placebo group (90 and 96.5 days vs. 50.5 days, respectively). Notably, the combination treatment further improved median survival to over 120 days. Furthermore, the combination group exhibited the highest percentage of complete remission, corresponding to a twofold increase compared to the placebo group. Importantly, none of the treatments induced significant nephrotoxicity. Additionally, the therapies affected various molecular targets involved in critical processes such as apoptosis, hypoxia and angiogenesis.

**Conclusion:**

In conclusion, the combination of VIP116 and [^177^Lu]Lu-DOTATATE presents a promising novel treatment approach for neuroblastoma. These findings hold potential to advance research efforts towards a potential cure for this vulnerable patient population.

**Supplementary Information:**

The online version contains supplementary material available at 10.1007/s00259-023-06462-3.

## Introduction

Neuroblastoma is a paediatric cancer form that originates from neural crest cells [1] and is characterised by a very heterogenous disease profile [2]. More than half of the cases are considered high risk, and treatments include surgery, external beam radiation therapy (EBRT), chemotherapy and retinoic acid. However, the survival prognosis for high-risk neuroblastoma remains around 50% [1].

One prominent issue with neuroblastoma is so-called micrometastases, also defined as minimal residual disease, which increase the risk of disseminated disease and potential future relapse [3]. Micrometastases are difficult to detect via conventional methods, and although significant advancements have been made in diagnostic methods [4], the same progress cannot be seen in treatment efficacy, resulting in an unmet clinical need for new and improved treatment strategies.

Molecular radiotherapy is a promising way to improve the treatment of several malignancies [5]. It allows for specific delivery of radiation to cancer cells while minimising damage to healthy tissue and is also highly suitable for localising and targeting occult, disseminated or micrometastatic disease [6]. Additionally, by using diagnostic radionuclides, one can quantify antigen expression, stratify patients and monitor and adjust pharmacokinetics and dosimetry by the use of nuclear medicine imaging techniques such as SPECT and PET [7], providing optimal conditions for molecular radiotherapy.

Recent advancements in cancer-targeting radiopharmaceuticals have revolutionised the field, boosting numerous clinical developments [5]. [^177^Lu]Lu-DOTATATE (Lutathera®) has been approved for the treatment of somatostatin receptor 2 (SSTR2) positive neuroendocrine tumours (NETs) by both EMA and FDA [8]. Between 75 and 90% of all clinical samples of neuroblastoma are considered positive for SSTR2 expression [9], and multiple early phase clinical trials have already shown promising results [10] using [^177^Lu]Lu-DOTATATE for treatment of neuroblastoma.

The p53 signalling pathway plays a crucial role in the response to radiation-induced DNA damage, by inducing cell cycle arrest or cell death [11], making it an attractive target for the potentiation of targeted radionuclide therapy [12]. The small stapled peptide VIP116 inhibits depletion of wildtype p53 (wt-p53) by inhibition of its negative regulators MDM2 and MDM4 [13]. Neuroblastoma exhibits very low rates of p53 mutations (mut-p53), making VIP116 a suitable treatment option, which multiple preclinical studies have confirmed [14]. VIP116 is yet to enter clinical trials, but another stapled peptide with inhibitory effects on MDM2/4, ALRN-6924, has been explored as a monotherapy for use against e.g. breast cancer. The drug exhibited a well-tolerable toxicity profile and a clear antitumoural response. However, this effect was not deemed consistent, and the development of ALRN-6924 as an anticancer agent was halted. It has since been investigated as monotherapy for use in paediatric cancer patients (ClinicalTrials.gov, NCT03654716) and as combination therapy with Paclitaxel (NCT03725436). It has also been investigated as a chemoprotective agent in several trials, but these trials were terminated due to haematological toxicity from the chemotherapy [15].

While [^177^Lu]Lu-DOTATATE therapy has shown great promise, challenges in the form of e.g. dose-limiting toxicities and insufficient or heterogenous target expression levels remain [16]. Neuroblastoma demonstrates high radiosensitivity, but for advanced disease presenting with metastatic lesions, the use of EBRT is not curative [17]. Combining [^177^Lu]Lu-DOTATATE and other treatment modalities could offer a more promising treatment option for advanced disease. Currently multiple options, including chemotherapy [18] and HSP90 inhibitors [19], are being explored as potential combination agents. By stabilising and upregulating wt-p53, VIP116 potentiates the effect of [^177^Lu]Lu-DOTATATE, by forcing the irradiated cells to undergo apoptosis more readily [20]. This could help overcome the challenge of insufficient target expression levels, as a lower radiation dose, and consequently a lower target expression, would be required to achieve a desirable effect. This combination could potentially also be of great benefit for patients with multiple lesions with varying target expression levels, reducing the risk of certain lesions responding while others progress. We have previously shown that the combination of [^177^Lu]Lu-DOTATATE and VIP116 displayed a synergistic inhibitory effect on the growth of neuroblastoma multicellular tumour spheroids [20], an in vitro model that can be used to mimic non-vascularised minimal residual disease [21], indicating that this may be a promising approach.

To summarise, while [^177^Lu]Lu-DOTATATE shows great promise for treatment of neuroblastoma, identifying combination strategies to overcome insufficient or heterogenous target expression and to lower radiation-induced toxicity is necessary and p53 stabilisation may provide a unique opportunity to potentiate radiotherapy effects in this patient group. Consequently, the aim of this study was to investigate the potential of the p53 stabiliser VIP116 and [^177^Lu]Lu-DOTATATE, both alone and in combination, in mice bearing human neuroblastoma xenografts. This study assessed effects on tumour growth, survival and toxicity, as well as underlying molecular mechanisms. To our knowledge, this is the first in vivo study of this promising combination strategy for treating neuroblastoma and will therefore provide valuable information to advance the research efforts towards a possible cure.

## Materials and methods

### Cell lines

IMR-32 and SK-N-AS, two human neuroblastoma cell lines, were purchased from American Type Culture Collection (Manassas, VA, USA) and cultured in MEM Earle’s (Biochrom, Berlin, Germany or Sigma-Aldrich, Darmstadt, Germany) and DMEM (Biowest, MO, USA), respectively. The murine neuroblastoma cell line Neuro 2a was purchased from Sigma-Aldrich (Darmstadt, Germany) and cultured in MEM Earle’s (Sigma-Aldrich, Darmstadt, Germany). All cells were cultured according to manufacturer’s instructions at 37 °C with 5% CO_2_ and passaged one to three times per week using 0.25% trypsin-EDTA (Life Technologies/Thermo Fisher, Waltham, MA, USA). All media was supplemented with 10% foetal bovine serum (Sigma-Aldrich, Darmstadt, Germany), 1% antibiotics (100 IU penicillin and 100 μg/mL streptomycin) and 1% L-glutamine (Biochrom, Berlin, Germany). In addition, the media for SK-N-AS and Neuro 2a also contained 1% non-essential amino acids (Thermo Fisher, Waltham, MA, USA). The in vitro SSTR2 expression as well as p53 mutational status was previously determined for IMR-32 (wild-type), Neuro 2a (mutated) and SK-N-AS (mutated) [20].

### Drug and radioconjugate preparation

Radiolabelling and quality control of [^177^Lu]Lu-DOTATATE was performed as previously described [20]. In brief, 1.5 μg DOTATATE (Bachem, Bubendorf, Switzerland) was mixed with 60 MBq of no-carrier-added (n.c.a.) [^177^Lu]LuCl_3_ (ITM, Munich, Germany) and incubated at 80°C for 30 minutes. Radiochemical yield of the non-isolated product was analysed with instant thin layer chromatography (ITLC) using a sodium citrate solution (0.1 M, pH 5.5) as the mobile phase. The average radiochemical yield was 95%. The dose administered to the animals in this study corresponds to the specific activity, i.e. the ^177^Lu conjugated to DOTATATE. Prior to injection, unlabelled DOTATATE was added in order to obtain a working concentration of 2 μg/mL in 0.9% NaCl.

A 10 mM stock solution of VIP116 in DMSO (produced by the p53 lab) was stored at −20°C [13]. For ^125^I-labelling, 2 µL of VIP116 (10 mM), 2 µL NaI (10 mM, MilliQ water) and 18 MBq of n.c.a [^125^I]NaI (PerkinElmer Inc., Boston, MA, USA) were incubated for 20 minutes at room temperature before the labelling reaction was initiated by addition of 15 µL chloramine-T (2 mg/mL, MilliQ water). The reaction was allowed to run for 60 seconds at room temperature before being quenched with 30 µL sodium metabisulfite (2 mg/mL, MilliQ water). The radiochemical yield was assessed with ITLC using 0.9% NaCl as mobile phase. The radiolabelling product was diluted in ethanol, and free ^125^I was removed using a PD Minitrap G10 column (GE Healthcare, Uppsala, Sweden), using ethanol as mobile phase.

### Xenograft establishment

All experiments complied with Swedish law and were performed with permission from the Uppsala Committee of Animal Research Ethics (Permit number: 10966/2020). Female BALB/c nu/nu mice (*N* = 73) from Charles River and female A/J mice (*N* = 6) from Envigo were housed under standard laboratory conditions and fed ad libitum. The mice were injected with either 10^7^ IMR-32 (BALB/c nu/nu, *N* = 63), 2*10^6^ SK-N-AS (BALB/c nu/nu, *N* = 2) or 2.5*10^6^ Neuro 2a (A/J, N = 6, BALB/c nu/nu, *N* = 8) cells subcutaneously on the hind leg. Mouse weight and tumour growth were monitored 1–3 times per week. Tumour diameter was measured using a digimatic caliper (Mitutoyo, Sweden), and volume was calculated as 4πabc/3 where a, b and c were measured diameters in all dimensions. A tumour volume equal to or above 1000 mm^3^ and/or weight loss of more than 10% from treatment start was defined as humane endpoints.

### Immunohistochemical analysis

To characterise the xenograft models, immunohistochemical analysis of SSTR2 expression on tumour tissue was performed. Untreated xenografts of IMR-32 (*N* = 6) , Neuro 2a (*N* = 3) and SK-N-AS (*N* = 2) were used. Furthermore, histological analysis of kidneys from animals taken 24 h (*N* = 7, *n* ≥ 2) or 6–11 weeks (*N* = 15, *n* ≥ 3) after last treatment was performed to assess toxicities related to the mono- and combination treatments. For more details on the treatments, see Therapy study section below.

All tissues were fixed in 4% buffered formalin, paraffin-embedded, sectioned and deparaffinised. Antigen retrieval was performed using low pH retrieval solution (Dako K8005, Agilent). Staining was performed using EnVision Flex Kit with DAB as chromogen and a Dako Autostainer 48 (Agilent, USA). Sections were immunostained with an antibody against SSTR2 (ab134152, Abcam, diluted 1/1000) and detected with Envision Flex kit (Dako K8010, Agilent). Counterstaining with haematoxylin (Histolab, Sweden) was performed in a Tissue-Tek Prisma (Sakura, Netherlands).

### Biodistribution of [^125^I]I-VIP116

To investigate the pharmacokinetics of subcutaneous injection of VIP116, a biodistribution of VIP116 in normal tissue was performed. [^125^I]I-VIP116 in ethanol was mixed with unlabelled VIP116 in 0.9% NaCl prior to injection to reach a VIP116 concentration of 200 μM. Final concentrations of ethanol and DMSO in the injection solution were 4.6% and 2%, respectively; 500 kBq (50 μL) [^125^I]I-VIP116 was injected subcutaneously on the right hind flank of BALB/c nu/nu mice (*N* = 6). After 24 (*n* = 4) or 48 (*n* = 2) h, mice were sacrificed, and the organs were collected, weighed, and the retained activity was measured using a gamma counter (1480 Wizard 3”, Wallac).

### VIP116 monotherapy

The therapeutic effect of VIP116 as monotherapy was assessed in BALB/c nu/nu mice bearing either IMR-32 (*N* = 19) or Neuro 2a (*N* = 8) tumour xenografts. The mice were treated with one injection per day for three consecutive days of either 50 μL of 200 μM VIP116 (*n* = 9 for IMR-32, *n* = 4 for Neuro 2a) or 0.9% NaCl containing 2% DMSO (*n* = 10 for IMR-32, *n* = 4 for Neuro 2a), subcutaneously above the tumour. Tumour volume and weight of the animals were monitored as described above.

### Biodistribution of [^177^Lu]Lu-DOTATATE

Once measurable tumours were established (1–2 weeks and 9 weeks post injection for Neuro 2a and IMR-32, respectively), 50 μL [^177^Lu]Lu-DOTATATE (2 μg/mL) was injected intravenously into the tail vein of BALB/c nu/nu mice bearing IMR-32 (*N* = 3) xenografts or A/J mice bearing Neuro 2a xenografts (*N* = 6). For Neuro 2a-bearing mice, the activity concentration was 10 MBq/mL. Biodistribution of mice bearing IMR-32 was performed using therapeutic doses of [^177^Lu]Lu-DOTATATE (80 MBq/mL). Animals were euthanised at 24h post injection, and a selection of organs were collected, including blood, tumour, kidneys and liver. The absorbed activity of each organ was then measured as described above.

### VIP116 and [^177^Lu]Lu-DOTATATE combination therapy study

Nine weeks after inoculation with IMR-32, animals were randomised in 4 groups (9–10 mice per group, including the 9 mice used for monotherapy, described above). Tumours were small (<50 mm^3^) or non-visible, and there was an equal distribution between visible and non-visible tumours in each group. Animals were treated with one intravenous injection of 50 μL 4 MBq [^177^Lu]Lu-DOTATATE (0.1 μg) diluted in 0.9% NaCl, one daily injection of 50 μL 200 μM VIP116 diluted in 0.9% NaCl (2% DMSO) on 3 consecutive days subcutaneously above the tumour, combination of both, or placebo; 0.9% NaCl and 2% DMSO in 0.9% NaCl were used as placebo for [^177^Lu]Lu-DOTATATE and VIP116 treatments, respectively. Data on mouse weight and tumour volume were collected as described above. Once animals had reached the endpoint (tumour volume of 1000 mm^3^), the animals were sacrificed, and tumours and kidneys were collected and fixed in formalin and serum samples were taken.

At study endpoint (120 days after treatment start), a [^177^Lu]Lu-DOTATATE biodistribution, as described above, was performed on all remaining animals. Visible tumours or the leg muscle at the original injection site as well as the leg muscle from the opposite leg were collected in order to compare uptake of [^177^Lu]Lu-DOTATATE and to determine if any unmeasurable tumour residue was present.

### Nephrotoxicity

Potential nephrotoxicity caused by treatment with [^177^Lu]Lu-DOTATATE and VIP116 was assessed by quantifying the percentage damaged glomeruli in immunohistology sections of the kidneys, as previously described [22]. In short, four 2-mm^2^ sections per animal were chosen, and the number of damaged and undamaged glomeruli in each section was determined and an average percentage for each animal was calculated.

Serum creatinine levels were determined using an enzymatic creatinine assay kit (MAK080, Sigma-Aldrich) according to the manufacturer’s instructions. In short, collected serum was first deproteinised using a 10 kDa MWCO spin filter (Vivaspin, Cytiva, Uppsala, Sweden), and the creatinine was enzymatically converted into a fluorescent end-product. The fluorescence intensity was measured (λex = 535 nm/λem = 587 nm) using a TECAN plate reader (210 Infinite series, TECAN, Switzerland), and the creatinine levels in the samples were calculated from a standard curve. The levels were then normalised against the mean fluorescence level in the untreated animals.

### Olink proteomic analysis

To assess the molecular effects of the treatments, spheroid culture lysates were analysed with Olink Proximity Extension Assay using the Oncology II panel (v.7004, Olink Biosciences), measuring expression of 96 proteins. IMR-32 spheroid lysates taken at 24 or 96 h post-treatment of [^177^Lu]Lu-DOTATATE, VIP116 or the combination of the two were prepared previously [20]. Protein levels were expressed as normalised protein expression (NPX) on a log2-scale.

Data analysis was performed with Matlab (R2022a, Mathworks). Principal component analysis (PCA) and hierarchical clustering were performed to analyse expression signatures between treatments. Prior to analysis, NPX values for each protein (assay) were normalised to Z-scores (mean=0, SD=1). PCA was performed using the *pca* function. Hierarchical clustering was performed on both samples and assays using the *pdist*, *linkage* and *optimalleaforder* functions.

To identify important proteins, a ranking system based on the standard deviation of each assay was used. A large standard deviation (big differences between treatments) corresponded to a high rank. This was performed on NPX values, normalised (by subtraction) to the control sample of the corresponding time point, with the *std* function.

### Visualisation and statistical analysis

Data was visualised and analysed using Prism 9 (GraphPad, 9.4.1) unless otherwise stated. NDP.view 2.9.29 (Hamamatru Photonics) was used for viewing and analysing the immunohistochemistry images. The final figures were created using InkScape (1.2.2).

## Results

### IMR-32, SK-N-AS and Neuro 2a express SSTR2 at varying levels

**Fig. 1:**
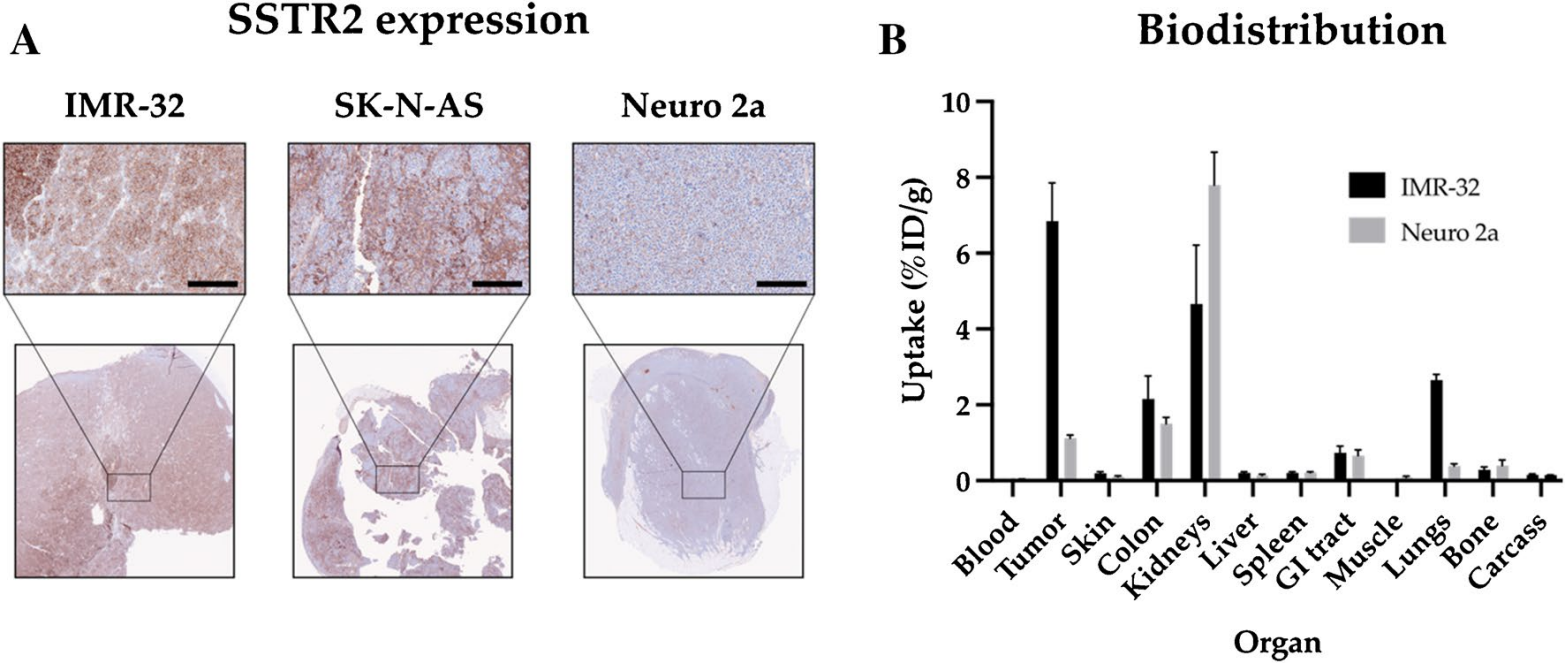
SSTR2 characterisation. **A** Ex vivo immunohistochemistry expression of somatostatin receptor 2 (SSTR2) in IMR-32, SK-N-AS and Neuro 2a tumour xenografts. Field of view = 0.9 mm^*2*^, bar = 100 μm. **B** Biodistribution at 24 h post-injection of [^177^Lu]Lu-DOTATATE in mice bearing either IMR-32 and Neuro 2a tumour xenografts. Uptake (%ID/g) is shown as mean ± SD, *n* ≥ 3

The ex vivo SSTR2 expression of IMR-32, SK-N-AS and Neuro 2a xenografts was examined using immunohistochemistry (Fig. 1A). All cell lines were positive for SSTR2 expression, with IMR-32 having the highest expression and Neuro 2a the lowest. For IMR-32 and SK-N-AS, there were also clear variations in staining intensity, with positive and negative subpopulations within one xenograft.

To further validate in vivo specificity and therapeutic potential of [^177^Lu]Lu-DOTATATE, biodistribution studies were performed in IMR-32 (high SSTR2 expressing cell line, BALB/c nu/nu mice) and Neuro 2a (low SSTR2 expressing cell line, A/J mice) xenografts (Fig. 1B). IMR-32 tumours displayed a mean tumour uptake of 6.8 %ID/g (SD ± 1.01) at 24 h post injection, while Neuro 2a only had a mean uptake of 1.1 %ID/g (SD ± 0.09). A similar biodistribution profile of [^177^Lu]Lu-DOTATATE in normal organs was seen for both xenograft models, with exception of kidneys and lungs. A lower renal uptake (4.7 %ID/g (SD ± 1.60) vs. 7.8 %ID/g (SD ± 0.87)), but higher pulmonary uptake (2.7 %ID/g (SD ± 0.15) vs. 0.39 %ID/g (SD ± 0.06)) was seen in IMR-32-bearing BALB/c nu/nu mice compared to Neuro 2a A/J mice.

### VIP116 monotherapy delays tumour growth in IMR-32, but not in Neuro 2a

**Fig. 2:**
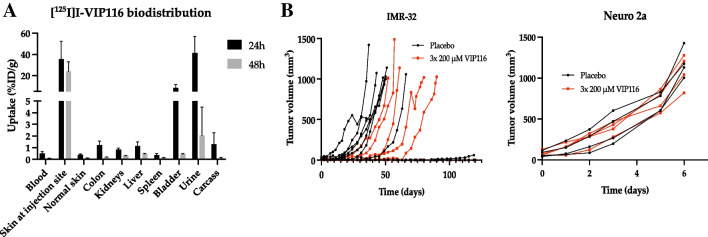
VIP116 in vivo characterisation. **A** Biodistribution of 500 kBq [^125^I]I-VIP116 at 24 and 48 h post-injection. Uptake (%ID/g) is shown as mean ± SD, *n* ≥ 2. **B** VIP116 monotherapy in mice with IMR-32 and Neuro 2a tumour xenografts. Graphs display the growth of individual tumours, *n* ≥ 4

The biodistribution and excretion of subcutaneously administrated [^125^I]I-VIP116 in normal tissue was investigated (Fig. 2A). The major sites for the radioconjugate at 24 h post-injection were the skin at the injection site, the bladder and urine, with only 0.5 %ID/g (SD ± 0.18) present in the blood. The mean uptake in the skin at the injection site was 35 %ID/g (SD ± 16.9) at 24 h post-injection, with 23.9 %ID/g (SD ± 9.29) still remaining at 48 h post-injection. The corresponding mean uptake in the skin of the opposite flank was 0.39 %ID/g (SD ± 0.08) at 24 h, and 0.11 %ID/g (SD ± 0.014) at 48 h.

A monotherapy study was performed on mice bearing either IMR-32 or Neuro 2a tumour xenografts (Fig. 2B). The IMR-32 tumours treated with VIP116 displayed a clear delay in growth in comparison to the untreated tumours, while Neuro 2a tumours were unaffected by the VIP116 treatment.

### [^177^Lu]Lu-DOTATATE and VIP116 combination therapy doubles complete remission

**Fig. 3:**
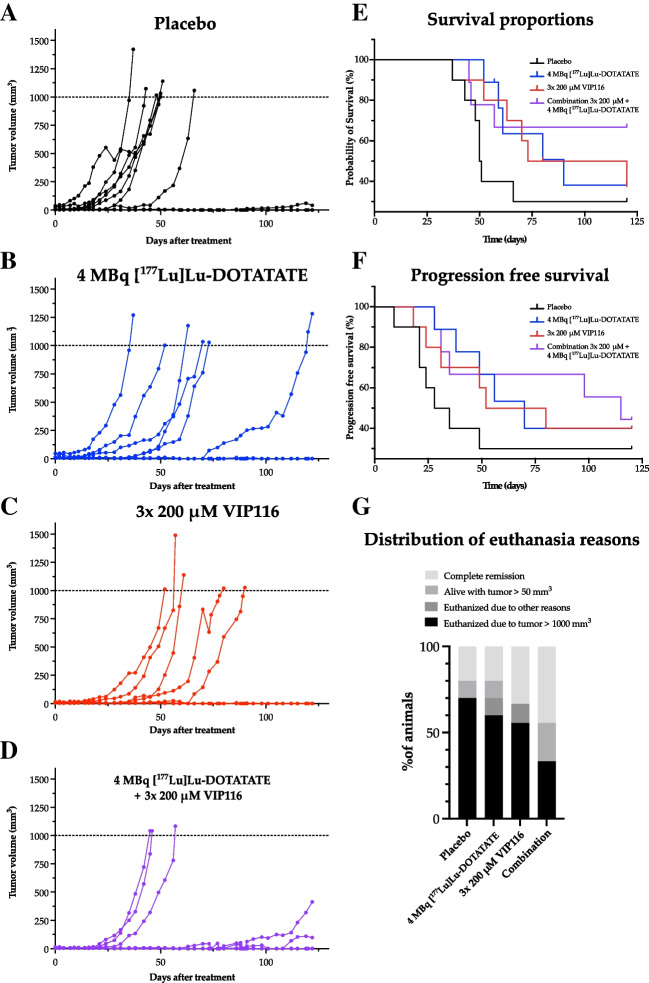
Combination therapy study. **A-D** Growth of individual IMR-32 tumours treated with placebo (***A***), 4 MBq [^177^Lu]Lu-DOTATATE (***B***), 3x 50 μL of 200 μM VIP116 (***C***) or the combination of both (***D***). *n* ≥ 9. **E** Kaplan-Meier curve displaying survival proportions for the different treatments, *n* ≥ 9. **F** Progression-free survival, defined as the time until tumour volume ≥ 100 mm^*3*^, for all treatment groups, *n* ≥ 9. **G** Distribution of euthanasia reasons amongst the treatment groups, *n* ≥ 9

The combination effect of VIP116 and [^177^Lu]Lu-DOTATATE was assessed in IMR-32 xenografts in mice (Fig. 3). The mice were treated with 4 MBq [^177^Lu]Lu-DOTATATE, one daily subcutaneous injection of VIP116 for 3 consecutive days, combination therapy, or placebo. Tumour growth varied between groups (Fig. 3A–D), with the placebo group showing the fastest growth (Fig. 3A). The monotherapy groups (Fig. 3B–C) exhibited a delay in tumour growth compared to the placebo group. The combination group showed the most significant delay, with the majority of the animals not reaching a tumour volume >500 mm^3^ (Fig. 3D). Median survival was shortest in the placebo group (50.5 days), while the monotherapies extended the median survival to 90 and 96.5 days for VIP116 and [^177^Lu]Lu-DOTATATE, respectively (Fig. 3E). At the study endpoint, the combination group had not yet reached the median survival, with the majority of the mice still alive. Progression-free survival (PFS), defined as time until tumour volume ≥100 mm^3^, displayed a similar pattern (Fig. 3F). The placebo group had a median PFS of 31.5 days, while monotreatment with [^177^Lu]Lu-DOTATATE or VIP116 extended the PFS to 66 and 70 days, respectively. The combination treatment resulted in a median PFS of 115 days.

At the end of the study, 17 animals were still alive (Fig. 3G). To determine if they had any residual disease and assess the number of complete remissions, a biodistribution with [^177^Lu]Lu-DOTATATE was performed (Table S1). The mice with measurable tumours all presented a significant difference in [^177^Lu]Lu-DOTATATE uptake between the tumour and the muscle of the opposite flank, whereas mice with no measurable tumour did not. At the study endpoint, 20% of all placebo mice had not developed a tumour, resulting in an estimated tumour take of 80%. In the [^177^Lu]Lu-DOTATATE and VIP116 groups, the number of mice without detectable tumours at study end was 20 and 33%, respectively. In the combination group, 44% had no detectable tumour, which is a 2-fold increase compared to the placebo group.

### Treatment with [^177^Lu]Lu-DOTATATE and VIP116 does not cause nephrotoxicity

**Fig. 4:**
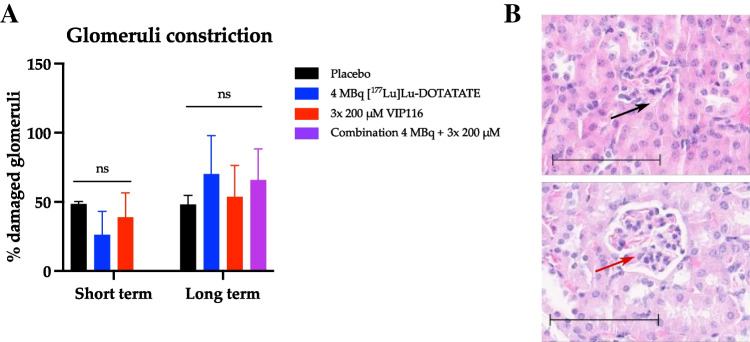
Nephrotoxicity. **A** Percentage of damaged glomeruli in kidneys belonging to animals treated with placebo (black), 4 MBq [^177^Lu]Lu-DOTATATE (blue), 3x 50 μL of 200 μM VIP116 (red) or the combination of the two (purple). Short term = 24 h after last treatment (*N* = 7, *n* = ≥ 2 per treatment group). Long term = at study endpoint, 120 days after treatment start (*N* = 15, *n* ≥ 3). **B** Representative images of a healthy glomeruli (black arrow) and damaged glomeruli (red arrow). Field of view = 0.028 mm^*2*^, bar = 100 μm

DOTATATE is a small peptide that undergoes renal excretion, and its reabsorption may result in nephrotoxicity when conjugated to ^177^Lu [23]. Potential nephrotoxicity from the treatment was determined by evaluation of glomeruli constriction in histopathological sections and by measurements of serum creatinine levels. Glomeruli constrictions were identified in all groups, albeit with no significant differences between untreated and treated animals (Fig. 4). This was in line with the serum creatinine measurements, which showed no significant differences in creatinine levels between the treatment groups or placebo after treatment (data not shown).

### Treatment affects molecular targets involved in apoptosis, hypoxia and angiogenesis

**Fig. 5:**
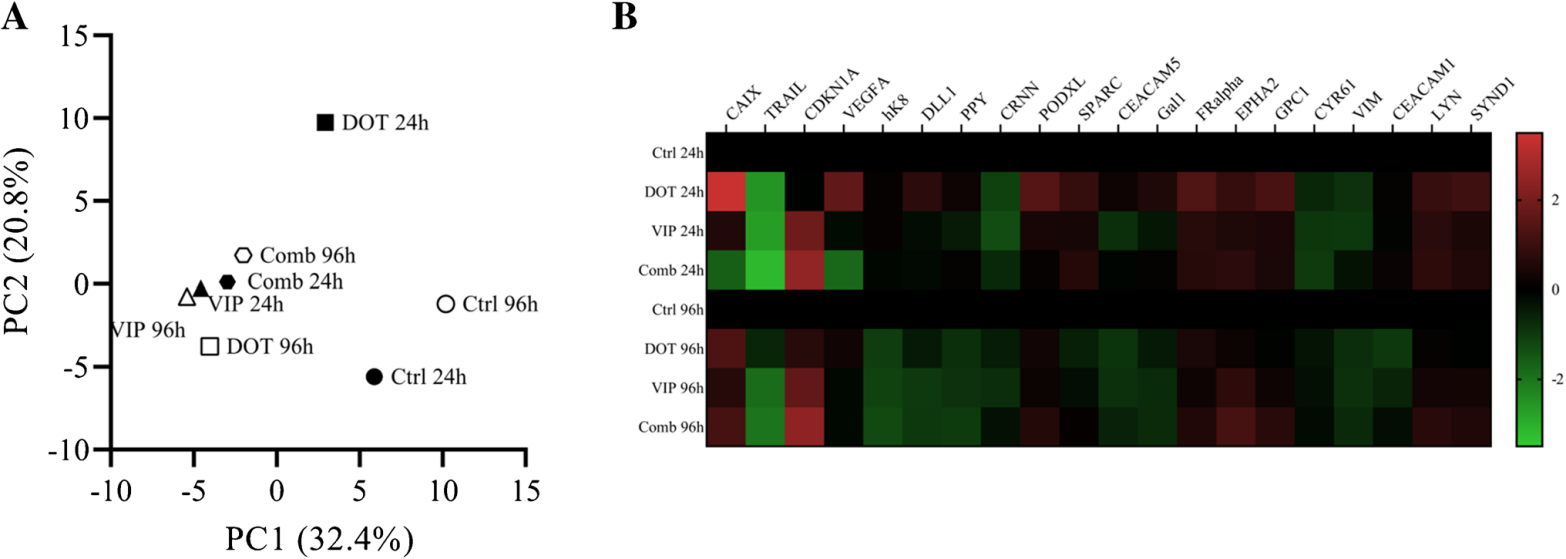
Olink proteomic analysis of IMR-32 spheroids. A Summary of PCA, displaying the two first principal components. Each data point represents one sample taken at 24 (black) or 96 (white) h after treatment. **B** Summary of the 20 highest ranked proteins. Each row represents one sample. Data is presented as NPX values, normalised to the control sample for each time points

Molecular effects of treatments on IMR-32 multicellular tumour spheroids were determined with proteomic analysis using Olink technology. PCA (Fig. 5A) and hierarchical clustering (Fig. S1) were used to compare expression signatures between treatments. The two first principal components of the PCA captured approximately 53% of the variation in the data set. These cluster all treated samples, except [^177^Lu]Lu-DOTATATE taken at 24 h after treatment, together. The untreated samples were further separated from the treated samples.

Ranking of each assay, based on standard deviation for each assay in the data set, was used to identify important proteins (Fig. 5B). Several different targets were affected by the treatment, including TRAIL, CDKN1A, CAIX and VEGFA. As expected, CDKN1A (p21) was upregulated in spheroids that had received either monotherapy or combination therapy with VIP116, but not in the control groups and monotreatment of [^177^Lu]Lu-DOTATATE. The hypoxia-related metalloenzyme carbonic anhydrase 9 (CAIX) was upregulated in the monotreatment of [^177^Lu]Lu-DOTATATE but downregulated in the combination group at 24 h. At 96 h, a slight upregulation was found in all treatment groups. A downregulation of tumour necrosis factor-related apoptosis-inducing ligand (TRAIL), a cytokine which mediates p53-independent apoptosis, was found in all treatment groups. VEGFA was upregulated at 24 h after monotreatment with [^177^Lu]Lu-DOTATATE, but downregulated in the combination group at the same time point. At 96 h, no difference between untreated and treated groups was found.

## Discussion

This study aimed to evaluate the in vivo effects of VIP116, a p53 stabiliser, and [^177^Lu]Lu-DOTATATE, a molecular radiotherapy, either alone or in combination for treatment of neuroblastoma. [^177^Lu]Lu-DOTATATE targets the SSTR2 receptor and has shown promising results in early clinical trials for neuroblastoma [10, 24]. VIP116, which stabilises wt-p53, is particularly suitable for treatment of neuroblastoma since it has a low p53 mutation rate [25]. The combination of p53 activation through radiation-induced DNA damage with inhibition of negative feedback on p53 from MDM2/4 could possibly result in an enhanced anti-tumour effect. Our previous study demonstrated a synergistic inhibitory effect of this combination treatment on neuroblastoma tumour spheroid growth, motivating further exploration in an in vivo setting [20]. This combination approach could also overcome challenges like insufficient or heterogenous target expression levels and allow for effective treatment at lower radiation doses, reducing the risk of radiation-induced toxicities and malignancies, which is of great importance in a paediatric population.

The study assessed three neuroblastoma xenograft models derived from two human (IMR-32 and SK-N-AS) and one murine (Neuro 2a) cell line. Studies were performed in neuroblastoma models designed to mimic minimal residual disease at time of treatment. Immunohistochemistry (IHC) confirmed the expression of SSTR2 in all three cell lines (Fig. 1A), with IMR-32 displaying the highest expression level, which is in line with previously obtained in vitro results [20]. IHC stainings identified the second highest expression in SK-N-AS tumours, followed by Neuro 2a, which is in contrast to previously obtained in vivo results [20], whereas SK-N-AS displayed no detectable uptake of [^177^Lu]Lu-DOTATATE. Changes in receptor expression when going from in vitro to in vivo settings could be a possible explanation behind these contrasting results [26].

Tumour uptake and normal organ distribution of [^177^Lu]Lu-DOTATATE was then assessed in IMR-32 and Neuro 2a xenografts (Fig. 1B). Mice bearing IMR-32 tumour xenografts demonstrated a mean tumour uptake of 6.8 %ID/g (SD ± 1.01) at 24 h post-injection, which is in line with previously reported levels in IMR-32 xenografts [27]. Neuro 2a xenografts displayed a mean tumour uptake of only 1.1 %ID/g (SD ± 0.09), which was in line with the IHC results and previously reported in vitro uptakes [20]. These results validated the in vivo antigen specificity of [^177^Lu]Lu-DOTATATE and further confirmed the suitability of IMR-32 xenografts for subsequent therapy studies with [^177^Lu]Lu-DOTATATE. A/J mice with Neuro 2a tumours displayed a higher renal uptake compared to BALB/c nu/nu mice with IMR-32 tumours. Conversely, lung uptake was higher in IMR-32-bearing mice. These variations may be due to anatomical differences between the two strains. For example, A/J mice are known to have small kidneys for their body weight [28].

VIP116 was evaluated for normal organ distribution and administration route through biodistribution of ^125^I-labelled VIP116. Subcutaneous injection led to drug accumulation in the skin at the injection site (Fig. 2A), remaining for an extended period of time. This aligns with previous research suggesting slower drug release with subcutaneous administration compared to intravenous delivery [29], supporting subcutaneous injection as a suitable route for this study. VIP116 exhibited a low uptake in normal tissues, in line with levels observed after intravenous injection of [^125^I]SIB-VIP116, an alternatively iodinated version of VIP116. This version has increased lipophilicity compared to the version used in this study, resulting in clearance by the liver instead of the kidneys, which was reflected in the biodistribution data. The peak uptake in SJSA-1 osteosarcoma tumour xenografts was 2.19 ± 0.56 %ID/g at 6 h post-injection, with only 0.36 ± 0.12 %ID/g remaining at 24 h [30]. In contrast, the uptake of subcutaneously injected [^125^I]I-PM2, the predecessor to VIP116, in HCT-116 colon cancer tumour xenografts displayed a tumour uptake of around 20 %ID/g at 24 h post-injection and a prolonged retention [31]. A subcutaneous injection bypasses systemic circulation, which could account for the higher tumour uptake. ALRN-6924, an MDM2/4 antagonist currently in clinical trials, is administered intravenously [32], but clinical use of subcutaneous injections for anti-cancer agents like Trastuzumab and Methotrexate [33] demonstrates the clinical applicability of this route. The most appropriate administration route for VIP116 in humans remains to be determined.

Monotherapy using VIP116 in mice bearing IMR-32 or Neuro 2a tumour xenografts confirmed the wt-p53 specificity of the drug (Fig. 2B). There was a clear delay in growth for the IMR-32 tumours (wt-p53), while the Neuro 2a tumours (mut-p53) were unaffected by the treatment.

To summarise, the initial characterisations demonstrated positive SSTR2 expression in all three in vivo NB-models, validated the p53 stabilising properties and wt-p53 specificity of VIP116 in vivo and identified IMR-32 as the most suitable xenograft model for subsequent [^177^Lu]Lu-DOTATATE studies.

Therapeutic effects of VIP116 and/or [^177^Lu]Lu-DOTATATE treatments were then assessed in IMR-32 xenografts. Median survival was increased for all treated mice, with the combination group showing the most significant increase, with its median survival not yet reached at the study endpoint (Fig. 3E). A [^177^Lu]Lu-DOTATATE biodistribution at study endpoint demonstrated that no detectable tumour residue remained in mice with no externally visible tumours, demonstrating an increased number of complete remissions in the combination group (Table S1). The obtained results demonstrate the potential of using VIP116 and [^177^Lu]Lu-DOTATATE for treatment of neuroblastoma, both as separate monotherapies and in particular in combination with each other.

Since [^177^Lu]Lu-DOTATATE is a small molecule that undergoes renal excretion, there is a concern for nephrotoxicity. Comparisons of glomeruli constriction in immunohistological sections of kidneys from untreated and treated mice showed no significant difference in number of damaged glomeruli between the groups (Fig. 4A). The percentage of damaged glomeruli in the untreated animals was higher than previously obtained results [22], possibly explained by the old age of the animals in the present study. No significant difference in serum creatinine levels between treatment groups were found (data not shown), further supporting the conclusion that the combination treatment did not significantly increase nephrotoxicity.

Treatment with VIP116 and [^177^Lu]Lu-DOTATATE resulted in the changed expression of several molecular targets. CDKN1A (p21), involved in the p53 signalling pathway, was upregulated in all VIP treatment groups, consistent with the proposed effect of VIP116 and previous findings [20]. At 24 h after treatment, CAIX, a pH-regulating enzyme and hypoxia marker, was significantly upregulated in the [^177^Lu]Lu-DOTATATE group [34]. CAIX can also influence the cellular response to radiation by preserving an alkaline pH in the cancer cell [35], which is possibly what is seen after treatment with [^177^Lu]Lu-DOTATATE in this study. In contrast, combination therapy resulted in downregulation of CAIX, which has been associated with increased cell death and improved radiation sensitivity [35], potentially explaining the enhanced therapeutic effects. TRAIL, a cytokine involved in p53-independent apoptosis [36], was downregulated in all treatment groups. Inhibition of MDM2 has been shown to promote TRAIL induced apoptosis in wt-p53 tumours [37], but studies have also shown that some neuroblastomas display resistance towards this [38]. Consequently, downregulation of TRAIL in treatment groups should not significantly affect cell death in the present setting. Instead, the growth inhibitory effects seen are more likely attributed to the p53 pathway, which is the main target for VIP116. More detailed studies of TRAIL signalling in regards to VIP116 and/or [^177^Lu]Lu-DOTATATE treatment are needed in order to fully understand these contrasting results. VEGFA expression was initially upregulated in the [^177^Lu]Lu-DOTATATE group but downregulated in the combination group. Ionising radiation has been shown to cause an upregulation of VEGFA expression [39], which could explain the upregulation in the [^177^Lu]Lu-DOTATATE monotreatment group. During prolonged hypoxia and genotoxic stress, e.g. in the form of radiation induced DNA damage, p53 will reduce the VEGFA expression in a p21 dependent manner [40]. This is in line with the observed results of an upregulation of both VEGFA and p21 in the combination group at 24 h. In summary, treatment with VIP116 and [^177^Lu]Lu-DOTATATE affects several molecular targets involved in processes including apoptosis, angiogenesis and hypoxia that in turn contribute to an anti-tumour effect.

To conclude, this study demonstrates for the first time the potential of p53 stabilisation as a tool to potentiate molecular radiotherapy, such as [^177^Lu]Lu-DOTATATE, in neuroblastoma in vivo models. Results demonstrated clearly delayed time to tumour progression, prolonged survival and a twofold increase in complete remissions in the combination group, with no signs of toxicity. Although more research is required, the results of this study show that this combination may be a potential game-changer in the future and could help to eradicate minimal residual disease and increase cure in neuroblastoma patients with confirmed SSTR2 expression and wt-p53 mutational status.

### Supplementary information

ESM 1 (DOCX 1428 kb)
