# Supplementary material for: p53 stabilisation potentiates [177Lu]Lu-DOTATATE treatment in neuroblastoma xenografts
Source: Eur J Nucl Med Mol Imaging. 2023 Oct 12;51(3):768–78. doi: 10.1007/s00259-023-06462-3 (PMC10796565; doi:10.1007/s00259-023-06462-3)
Supplement: Supplementary file 1 — (DOCX 1428 kb) [file 259_2023_6462_MOESM1_ESM.docx]

**p53 stabilisation potentiates [^177^Lu]Lu-DOTATATE treatment in neuroblastoma xenografts**

**Authors and affiliations**

Hanna Berglund^1†^, Sara Lundsten Salomonsson^1,2†^, Tabassom Mohajershojai^1^, Fernando Jose Ferrer^3^, David P. Lane^1,3,4^ & Marika Nestor^1^

^1^ Department of Immunology, Genetics and Pathology, Uppsala University, SE-751 85 Uppsala, Sweden

^2^ Ridgeview Instruments AB, SE-752 38, Uppsala, Sweden

^3^ p53Lab, Agency for Science Technology and Research (A*STAR), Singapore 138648, Singapore

^4^ Department of Microbiology, Tumour and Cell Biology, Karolinska Institute, SE-171 65 Solna, Sweden

^†^ Equal contribution

**Correspondence:** Marika Nestor, [marika.nestor@igp.uu.se](mailto:marika.nestor@igp.uu.se)

**Table S1. Final biodistribution.** Table showing the difference in uptake (%ID/g) of [^177^Lu]Lu-DOTATATE between tumor/muscle at implantation site and the muscle from the opposite flank. N=17.


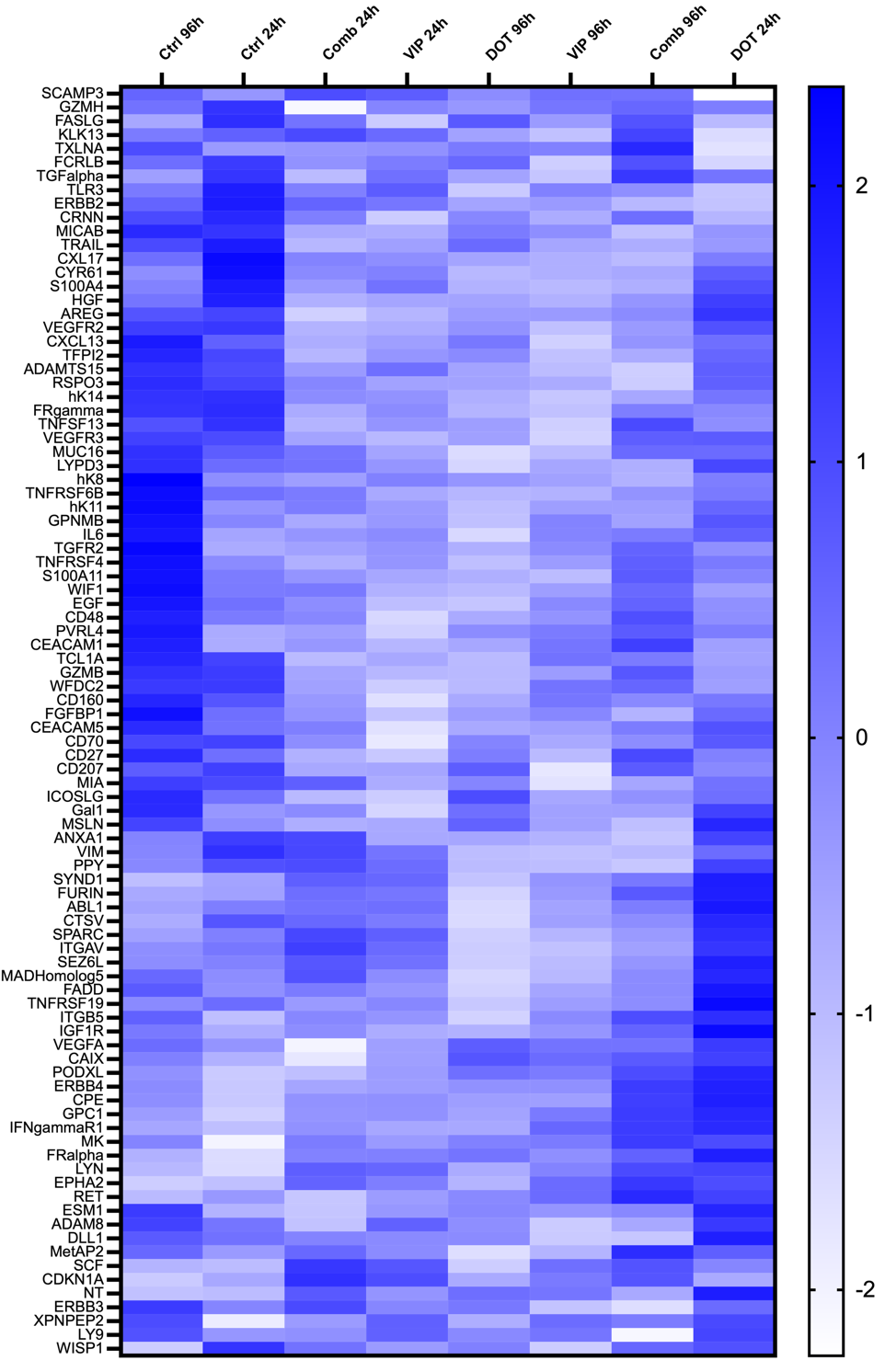


**Figure S2. Summary of Olink data.** Z-scores of all measured 92 proteins. Hierarchical clustering was performed on both targets and samples.
